# Supplementary material for: Investigating the influence of physiologically relevant hydrostatic pressure on CHO cell batch culture
Source: Sci Rep. 2021 Jan 8;11:162. doi: 10.1038/s41598-020-80576-8 (PMC7794228; doi:10.1038/s41598-020-80576-8)
Supplement: Supplementary file 1 — Supplementary Information 1. [file 41598_2020_80576_MOESM1_ESM.zip › S1/IgG/Feb17 60 90 standard/old/Feb15 60mmHg 3.pdf]

Data File C:\Users\P...mStation\1\Data\TK_CHO_IgG 2019-02-17 14-04-01\030-4-60mmHg Feb15.D
Sample Name: 60mmHg Feb15

===

Acq. Operator	: SYSTEM	Seq. Line :	30
Sample Operator	: SYSTEM		
Acq. Instrument	: HPLC-0039	Location :	4
Injection Date	: 2/17/2019 4:09:14 PM	Inj :	1
		Inj Volume :	100.000 µl

Method : C:\Users\Public\Documents\ChemStation\1\Data\TK_CHO_IgG 2019-02-17 14-04-01 \CHO_IgG_LC.M (Sequence Method)
Last changed : 2/8/2019 3:17:23 PM by SYSTEM

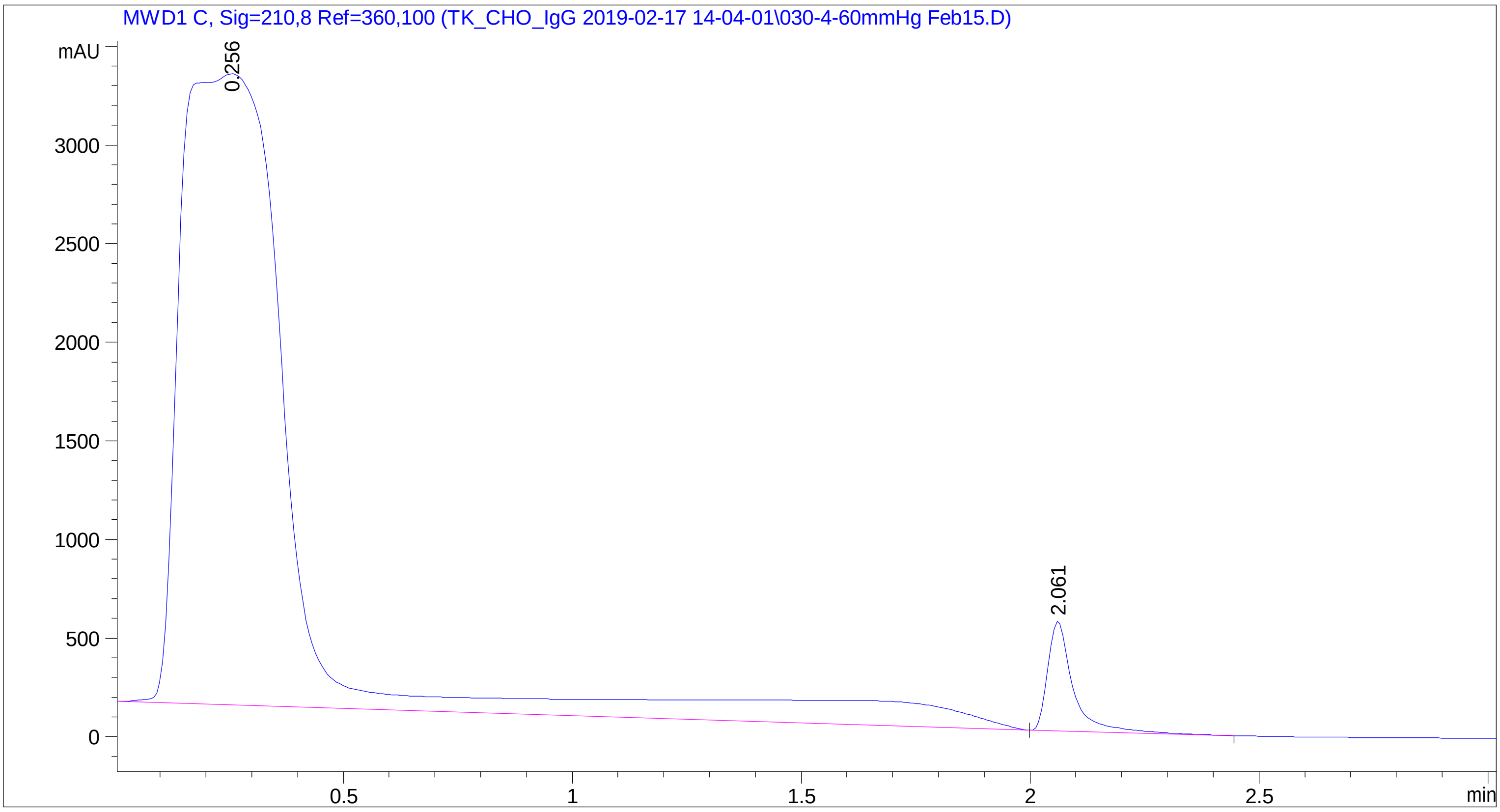

===
Area Percent Report
===

Sorted By	:	Signal
Multiplier	:	1.0000
Dilution	:	1.0000

Use Multiplier & Dilution Factor with ISTDs

Signal 1: MWD1 C, Sig=210,8 Ref=360,100

Peak #	RetTime [min]	Type	Width [min]	Area [mAU*s]	Height [mAU]	Area %
1	0.256	BB	0.2363	5.43951e4	3199.59668	96.2296
2	2.061	BBA	0.0587	2131.25391	556.68231	3.7704

Totals :	5.65263e4	3756.27899

===
*** End of Report ***
